# Timing matters: using optogenetics to chronically manipulate neural circuitry and rhythms

**DOI:** 10.3389/fnbeh.2014.00041

**Published:** 2014-02-14

**Authors:** Michelle M. Sidor, Colleen A. McClung

**Affiliations:** Department of Psychiatry, University of Pittsburgh School of MedicinePittsburgh, PA, USA

**Keywords:** optogenetics, opsins, circadian rhythms, addiction, depression, bipolar disorder, obsessive-compulsive disorder, mouse models

## Abstract

The ability to probe defined neural circuits with both the spatial and temporal resolution imparted by optogenetics has transformed the field of neuroscience. Although much attention has been paid to the advantages of manipulating neural activity at millisecond timescales in order to elicit time-locked neural responses, little consideration has been given to the manipulation of circuit activity at physiologically relevant times of day, across multiple days. Nearly all biological events are governed by the circadian clock and exhibit 24 h rhythms in activity. Indeed, neural circuit activity itself exhibits a daily rhythm with distinct temporal peaks in activity occurring at specific times of the day. Therefore, experimentally probing circuit function within and across physiologically relevant time windows (minutes to hours) in behaving animals is fundamental to understanding the function of any one particular circuit within the intact brain. Furthermore, understanding how circuit function changes with repeated manipulation is important for modeling the circuit-wide disruptions that occur with chronic disease states. Here, we review recent advances in optogenetic technology that allow for chronic, temporally specific, control of circuit activity and provide examples of chronic optogenetic paradigms that have been utilized in the search for the neural circuit basis of behaviors relevant to human neuropsychiatric disease.

## Introduction

Optogenetics has transformed the field of neuroscience with its ability to manipulate neural circuit activity with unprecedented spatial and temporal precision. Indeed, the ability to manipulate neural activity at physiologically relevant millisecond timescales has leveraged an advantage of using optogenetics as a tool to link time-locked changes in neural activity to behavioral and/or physiological events and has been instrumental in our understanding of the neural circuitry driving an array of behavioral states (Tye and Deisseroth, 2012; Nieh et al., 2013). Little consideration, however, has been given to (1) the importance of proper timing for delivery of optogenetic stimulation, (i.e., at physiologically relevant times of day); and (2) to the overall duration of manipulation (acute vs. chronic). Although treated separately for the purpose of this review, these two temporal considerations are ultimately interconnected: in addition to acute optogenetic stimulation, the expanding use of chronic stimulation paradigms, in general, will require attention to the circadian timing of circuit manipulation; the proposed use of optogenetics to alter circuit rhythms will require extended stimulation parameters.

## Consideration of diurnal-specific control of neural activity

**Figure 1:**
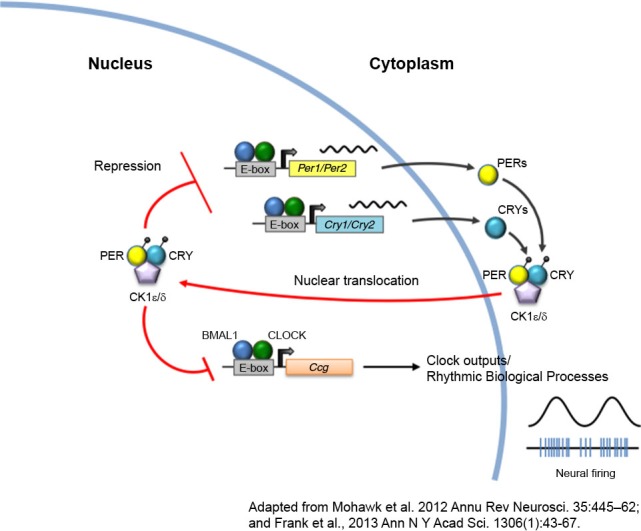
**The circadian clock machinery**. Twenty-four hour rhythms are generated by a cell-autonomous and autoregulatory transcriptional-translational feedback loop. In its simplest form, the core circadian protein, CLOCK, forms a complex with brain and muscle ARNT-like protein 1 (BMAL1). This complex binds to enhancer box (E-box) regulatory element and activates transcription of the *Period (Per)* and *Cryptochrome* (*Cry*) genes. As PERs and CRYs accumulate during the day, they dimerize and translocate back to the nucleus where they interact with CLOCK and BMAL1 to repress their own transcription. As these negative elements are degraded by casein kinases (CK) at night, repression of CLOCK and BMAL1 is removed, and a new cycle of transcription begins the following morning. The CLOCK-BMAL1 complex also binds to an array of clock-controlled genes (*ccg*) that ultimately regulate biological processes such as sleep-wake cycles, body temperature, hormone secretion, feeding, and activity.

**Figure 2:**
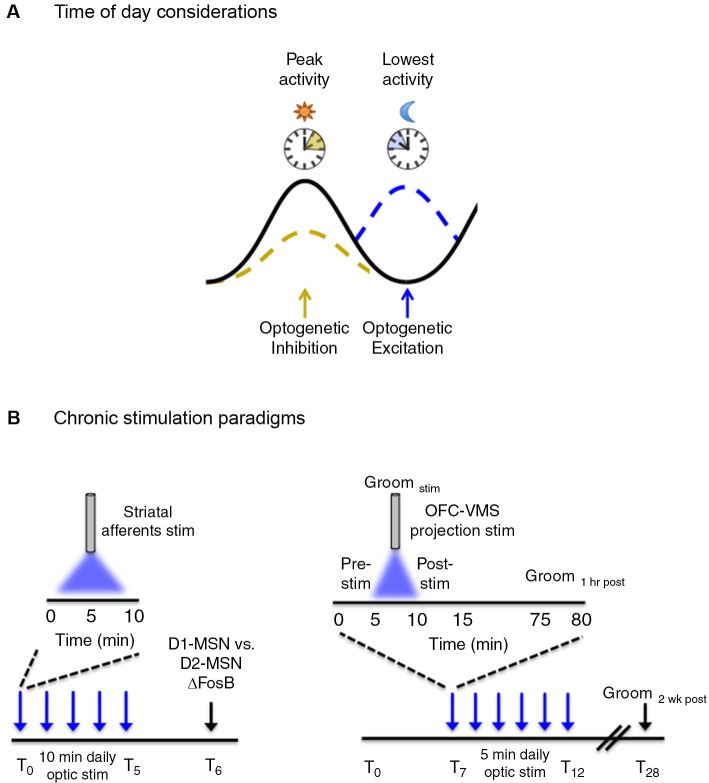
**Temporal considerations for optogenetic manipulation of neural activity.** **(A)** Neural activity exhibits natural daily rhythms with distinct peaks and troughs of activity emerging throughout the 24 h light/dark cycle. Neural circuit manipulation during these peaks and troughs may yield more pronounced behavioral effects, i.e., inhibition of a given circuit when activity is usually highest may be preferred over inhibiting when activity is at a daily low. This is a generalized concept and the converse may be equally as valid depending on the type of experiment performed and the purpose of optogenetic manipulation. **(B, left)** ΔFosB induction, a marker of chronic neural activity, was measured in striatal dopamine rececptor-1 (D1) and dopamine receptor 2 (D2) medium spiny neurons (MSNs) following five consecutive days of optogenetic stimulation of ChR2-expressing neurons in the following striatal afferents: medial prefrontal cortex (mPFC), amygdala, ventral hippocampus and the VTA. Daily optic stimulations involved 10 min bouts of blue light delivered at 20 Hz, 40 ms for phasic VTA stimulation and at 20 Hz for 30 s for prefrontal cortex, amygdala, and hippocampal stimulation. **(B, right)** Following 7 days of habituation to the tethering protocol, six consecutive days (*T*_7_–*T*_12_) of optogenetic stimulation were carried in awake freely-moving mice that involved daily 5 min bouts of stimulation with blue light, pulsed at 10 Hz (10 ms pulse width). Light was delivered to ChR2 expressing neurons in the orbitofrontal cortex (OFC) to ventromedial striatal (VMS) projection. Grooming behavior (as a measure of obsessive-compulsive-like behavior) was measured before (pre-stim), during (Groom_stim_), and after (post-stim) optic stimulation. Acute behavioral responses were assessed immediately following stimulation and 1 h post-stimulation. Chronic behavioral responses were assessed during the pre-stim period the following day, as a “24 h time-stamp” of previous daily stimulations. The persistence of the chronic stimulation protocol was assessed 2 weeks (*T*_28_) following the last day of stimulation.

The circadian clock is a temporal interface that synchronizes internal physiological and behavioral events to the external environment. A core group of proteins generate biological rhythms in an approximate 24 h cycle (Figure 1). The master clock governing these rhythms resides in the suprachiasmatic nucleus (SCN) of the anterior hypothalamus (Ralph et al., 1990) and coordinates the activity of self-sustained peripheral clocks (Yoo et al., 2004) that are expressed almost ubiquitously throughout bodily tissue. Components of the molecular clock are also found in extra-SCN regions in the central nervous system and notably in brain regions implicated in mood-regulation and reward, such as the hippocampus, amygdala, prefrontal cortex, lateral habenula, nucleus accumbens, and ventral tegmental area (VTA) of both rodents (Abe et al., 2002; McClung et al., 2005; Guilding and Piggins, 2007; Webb et al., 2009) and humans (Li et al., 2013). In addition to exhibiting rhythms in circadian gene expression, these brain regions exhibit rhythms in neural firing, neural activity, neurotransmitter levels and receptor expression (Guilding and Piggins, 2007; Sleipness et al., 2007; Hampp et al., 2008; Webb et al., 2009; Baltazar et al., 2013). Perceptible temporal peaks and troughs in neural activity are observed across brain regions with each displaying a unique temporal profile of activity. It is proposed that coordinated neural activity across regions is important for the proper daily timing of behaviors and avoids co-occurrence of conflicting motivational states (i.e., feeding vs. sleeping, for example). Therefore, consideration of not only how neural circuit activity impacts behavior but also how the timing of stimulation influences a given behavioral state may yield additional, ethologically relevant information. This is particularly relevant when studying the reward system as there is known daily variation in reward-seeking and drug response in both rodents and humans. For instance, the reinforcing properties of cocaine are greater during the light than dark-phase of the light/dark cycle in rodents (Abarca et al., 2002). Furthermore, nicotine administration and sensitivity in rodents and humans follows a daily rhythm, with more intense periods of administration and sensitivity to nicotine presenting during the light-cycle (Mooney et al., 2006; Mexal et al., 2012). Importantly, the propensity to administer drugs of abuse and the sensitivity to natural reward in rodents has been linked to diurnal changes in dopaminergic activity within the mesolimbic system (Webb et al., 2009). Therefore, neural circuit manipulation during peaks and troughs of activity may yield more pronounced behavioral responses, i.e., inhibition of a given circuit when activity is usually highest may be preferred over inhibiting when activity is at a daily low (Figure 2A). Alternatively, for long-term stimulation paradigms (discussed in next section), stimulating a circuit at a time of day that is not in accordance with its natural rhythm may disrupt overall network synchrony, which may or may not be the intended experimental effect.

## Diurnal rhythm changes will necessitate chronic stimulation paradigms

Conversely, one may want to use optogenetic approaches to purposefully disrupt or alter diurnal rhythms. Studies that have employed jet lag paradigms or other types of phase resetting protocols have demonstrated that days to weeks are required for an animal to fully adjust/entrain to a new light/dark cycle (Albrecht, 2012). It stands to reason, therefore, that studies aimed at altering the amplitude, phase and/or period of neural activity rhythms will require long stretches of time. This will necessitate the development of novel stimulation paradigms that permit both chronic excitation and inhibition within the same neurons. The prospect of these studies are exciting as they will directly determine the relevance of diurnal rhythms in select neuronal populations to disease states and basic brain function.

To exemplify this point, our lab uses the *ClockΔ19* mutant mouse to study how chronic alteration in the daily activity of specific neural circuits impacts reward and mood-related behaviors. These mice display a behavioral profile that is strikingly similar to human bipolar-mania (Roybal et al., 2007) that occurs within the overall context of disrupted circadian rhythms, driven by a point mutation in exon19 of the *Clock* gene (Vitaterna et al., 1994; King et al., 1997). Behavioral abnormalities include lower levels of anxiety-like behaviors, decreased depressive-like behaviors, hyperactivity, reduced sleep time, and increased propensity for both natural (i.e., sucrose) and drug rewards (Naylor et al., 2000; Roybal et al., 2007). Lithium treatment, which is used as a mood-stabilizing agent to treat human bipolar disorder, is also effective at reversing many of these behavioral abnormalities (Roybal et al., 2007). *ClockΔ19* mutant mice exhibit gross neural circuit abnormalities that relate to improper timing of neural events, including altered phase-coupling within the nucleus accumbens (Dzirasa et al., 2010) and deficits in synchronization of neural activity across limbic brain regions (Dzirasa et al., 2011). Additionally, *ClockΔ19* mice exhibit profound alterations in the diurnal activity of the mesolimbic dopamine system, (McClung et al., 2005; Spencer et al., 2012) which has been shown to underlie components of their manic-related behavioral profile (Coque et al., 2011). Although it is intriguing to speculate that altered circadian clock machinery is the driving force generating these neural circuit timing-deficits, the exact mechanism has yet to be fully elucidated. Regardless, the important point to emphasize is that manipulating the timing, in addition to the direction, of neural circuit activity is instrumental to understanding how altered neural activity contributes to abnormal behaviors. This same principle can be applied to the study of any behavior that exhibits natural daily rhythms and to the host of chronic disorders that have been linked to disruptions in biological rhythms (Bass and Takahashi, 2010; Yu and Weaver, 2011; Albrecht, 2012; McCarthy and Welsh, 2012; Coogan et al., 2013; Frank et al., 2013).

## Chronic optogenetic modulation of neural circuit activity

In general, understanding how chronic disruption of neural activity, i.e., that which occurs over the course of many days and weeks, contributes to the development of abnormal behavior may better model the chronic circuit-wide disruptions underlying neurological disorders, psychiatric disorders, and drug addiction. One can speculate that perturbing neural circuit activity chronically may induce neural plastic changes, such as altered synaptic structuring, altered neural firing properties, and overall network re-wiring, not induced by acute modulation. Chronic neural circuit modulation, therefore, has the potential to provide additional information not revealed by studying acute time-locked neural responses. Chronic optogenetic stimulation paradigms—defined as manipulation that occurs across multiple, consecutive days—have been used by relatively few studies. Their use, as demonstrated below, can often yield distinct results from those obtained using acute stimulation protocols. It should be noted that although we focus on the *in vivo* use of chronic manipulation, there are a few *in vitro* studies that have emerged which use chronic optogenetic stimulation for various applications (Stroh et al., 2011; Lignani et al., 2013).

In the first study, Lobo et al. (2013) used a chronic optogenetic stimulation protocol to determine the circuit-level mechanisms underlying distinct patterns of protein expression induced in striatal neurons in response to chronic stimuli (Figure 2B). Chronic stimuli such as drugs of abuse, stress, and natural reward lead to a stable up-regulation of the transcription factor, ΔFosB, in the striatum (McClung et al., 2004). This increase is thought to be important for the long-term adaptive changes associated with chronic stimulus exposure. Previous evidence has suggested that specific chronic stimuli differentially induce ΔFosB in specific subsets of striatal GABAergic projection medium spiny neurons (MSNs) (McClung et al., 2004; Pitchers et al., 2013). However, the specific circuits mediating the distinct induction of striatal ΔFosB in response to different stimuli were unknown. Lobo et al. (2013) used a chronic optogenetic approach to stimulate specific striatal afferents and examined the resulting pattern of ΔFosB induction in dopamine receptor 1 (D1) vs. dopamine receptor 2 (D2) enriched MSNs. To accomplish this, the light-sensitive ion channel, channelrhodopsin (ChR2), was expressed in the VTA and in glutamatergic neurons of the ventral hippocampus, medial prefrontal cortex (mPFC), and amygdala, followed by fiber optic implantation at the respective cell bodies. Mice received phasic pulses of blue light at 20 Hz for 40 ms to stimulate VTA neurons and 20 Hz pulses for 30 s for stimulation of mPFC, amygdala, and hippocampal neurons. Optic stimulation was delivered for 10 min a day for five consecutive days and ΔFosB was measured in D1 and D2 enriched MSNs 24 h following the last day of the 5 day stimulation protocol. Chronic optic stimulation of the different striatal inputs lead to a predominate induction of ΔFosB in D1-MSNs, consistent with previous studies showing that optogenetic stimulation of these afferents promote reward. Additionally this demonstrated that repeated episodes of 10 min optic stimulation were sufficient to induce neural plasticity similar to repeated exposure to drugs of abuse and natural rewards. The ability to probe cell-defined and projection-specific circuits leverages an advantage for using chronic optogenetic stimulation to delineate the circuit mechanisms driving adaptive neural changes.

In another study, Ahmari et al. (2013) addressed the role of cortico-striatal dysregulation in obsessive-compulsive disorder (OCD). There is compelling evidence to suggest a role for hyperactivity of the orbitofrontal cortex (OFC) and ventromedial striatum (VMS) in OCD pathology (Pittenger et al., 2011), however, a causal role has been difficult to ascertain. Ahmari and colleagues employed a chronic optogenetic stimulation protocol to directly assess the role of repeated cortico-striatal stimulation on OCD-like behaviors in mice (Figure 2B). To accomplish this, a cre-dependent virus encoding ChR2 was transduced into glutamatergic neurons of the OFC followed by a fiber optic implant in the VMS to chronically stimulate the OFC-VMS projection pathway. EMX-cre mice (ensures glutamate cell-type specificity for viral transduction) received 10 Hz, 10 ms pulses of blue light for 5 min a day, across multiple days. A sustained, stimulation-independent increase in OCD-like behavior (as indicated by excessive grooming relative to control mice) was observed following 3 days of stimulation. Repeated stimulation also led to a progressive increase in light-evoked VMS neural firing that paralleled the increase in repetitive grooming behavior. This effect persisted for 2 weeks after cessation of a 6 day stimulation protocol, indicating that chronic stimulation induced long-term plastic changes at OFC-VMS synapses. Importantly, changes in grooming behavior were not seen following acute stimulation, defined as grooming measured both immediately and 24 h following a single 5 min stimulation session. This indicates that chronic circuit changes are required for the expression of specific behaviors that extend beyond immediate changes in neural firing.

It should be emphasized that the opsins, or light-sensitive ion channels, used to transduce optic signals to neurons can vastly affect the permitted time scales used for stimulation. For instance, the work described above relied on ChR2 for control of neural activity, which is an opsin with fast channel photokinetics (Nagel et al., 2003; Boyden et al., 2005). This means that the ion channel opens and closes on a millisecond timescale in response to light and that repeated pulses of light are required for prolonged and sustained activation. This can become problematic as ChR2 has the potential to desensitize with repeated stimulation (Nagel et al., 2003), vastly limiting its use for protocols that involve prolonged periods of stimulation (i.e., over tens of minutes). Furthermore, repeated stimulation with light irradiances (mW/mm^2^) required to activate ChR2 may raise concern over the potential for significant heat generation, which can have deleterious effects on brain tissue (Yizhar et al., 2011a). Moving forward, the use of optogenetics for more prolonged stimulation will require opsins that do not readily desensitize and that are more sensitive to lower powers of light (such as the step-function opsins) (Berndt et al., 2009; Yizhar et al., 2011b).

## Technological advances permitting chronic optogenetic stimulation

There are a growing number of hardware options available that are compatible with chronic optogenetic stimulation protocols. For instance, chronic fiber implants have greatly reduced tissue damage associated with the repeated removal and insertion of optical fibers to deep brain structures. These optical neural interfaces include a fiber optic, which transmits light to deep brain structures, and a metal or ceramic ferrule that is secured to the fiber optic (Aravanis et al., 2007). The ferrule protrudes from the head for coupling and tethering to an external light source. These fiber cannulas are relatively simple to make in-house (Sparta et al., 2012) and can be surgically implanted and secured for chronic use (Ung and Arenkiel, 2012).

When the duration of the chronic stimulation protocol extends from minutes to hours and across many days, however, there are other hardware considerations that need to be made. For instance, animals are usually tethered to a light source during acute stimulation, however, more complex chronic stimulation protocols may involve substantially extended durations of time whereby prolonged tethering in the homecage or experimental environment is not possible. Furthermore, for multiple stimulations across a 24 h period, the constant removal and re-tethering of the animal may induce profound handling stress and have a negative impact on behavior. In these cases, wireless optogenetic technology may provide a truly remote and non-invasive system for chronic control of neural activity and will permit more complex chronic stimulation paradigms across a single day or multiple days and weeks.

Although the field of wireless optogenetic technology is still in its infancy, a variety of promising platforms have emerged that use infrared or radiofrequency (RF) signaling to remotely control a head-mounted device. This device can detect and relay wirelessly generated signals to light-emitting diodes (LEDs) that are secured directly on the head-mounted device (Iwai et al., 2011; Wentz et al., 2011; Ameli et al., 2013). This circumvents the need for tethered fiber optic devices that require a physical fiber cable connection between an animal and an external light source. Wireless head-mounted LEDs have been used to stimulate surface brain structures, such as the motor cortex in Thy1-ChR2 transgenic mice (Iwai et al., 2011), and have been coupled to implanted fiber optic cannulas for optic stimulation of deeper brain structures (Wentz et al., 2011). With the advent of red-shifted opsins, such as C1V1 (Yizhar et al., 2011b; Mattis et al., 2012) and the newer ReaChR (Lin et al., 2013) designed by Roger Tsien’s group, deep brain structures can be targeted wirelessly without the need for a fiber optic insert into the brain. In this case, a head-mounted red LED would be sufficient to activate opsins expressed in deep brain structures, as red light scatters less than blue light and can penetrate into deep tissue more readily.

Micro-LEDs offer a slightly different approach to wireless control of neural activity whereby a μ-LED is implanted directly into the brain rather than being mounted on a head-stage (Kim et al., 2013; McAlinden et al., 2013; McCall et al., 2013). At least one group has combined these cellular-scale injectable μ-LEDs with a head-mounted antenna for remote and wireless control of the chronically implanted μ-LEDs (Kim et al., 2013; McCall et al., 2013). The implanted LEDs were well tolerated by freely moving animals and maintained operational functionality up to the tested maximum of 6 months (Kim et al., 2013). As a proof of principle for wireless control of neural activity, the authors connected the implanted μ-LED probe to a head-stage antenna to detect wirelessly generated RF signals. VTA dopamine neurons transduced with a cre-dependent ChR2 were tonically stimulated using this wireless system. Here, tyrosine hydroxylase (TH): Cre mice received 5 Hz, 5 ms pulses of blue light for 3 min while behaving in the elevated zero maze—a validated behavioral test used to assess anxiety-like behaviors. Acute tonic stimulation of dopamine neurons produced an anxiolytic behavioral response, independent of locomotor changes, as measured while animals were being actively stimulated.

Many of these head-mounted devices, however, are bulky and would benefit from scaling-down to sizes compatible with long-term use in rodents in order to avoid damage to the hardware components. Furthermore, it is essential that steps be taken to ensure adequate detection of wirelessly generated signals as limited detection ranges/distances may present a current limiting factor. We look forward to continued advances in the fields of electrical, mechanical, optical, and biological engineering as this will lead to a vast expansion of technological options permitting novel and more refined ways to probe the nervous system (Deisseroth and Schnitzer, 2013).

## Summary

Here we propose that the time of day and duration (acute vs. chronic) of circuit modulation are important experimental considerations for certain optogenetic applications. Manipulation of neural activity at physiologically-relevant times of day is an important methodological consideration for both acute and chronic stimulation of circuits that display natural daily rhythms. Experiments aimed at altering diurnal rhythms using optogenetics will necessitate the use of chronic stimulation paradigms due to the extended timescale required for the entrainment of rhythms. Chronic stimulation paradigms have begun to emerge with the potential to uncover the neuroplastic events and circuit-wide disruptions associated with chronic disease states. Although these temporal considerations may not apply in all cases, an awareness of their rationale, experimental advantage, and appropriateness to specific experimental methodologies will permit more physiologically relevant paradigms for probing neural circuit function in health and disease.

## Conflict of interest statement

The authors declare that the research was conducted in the absence of any commercial or financial relationships that could be construed as a potential conflict of interest.
